# Efficacy of Leap Motion Device on Fine Motor Function and Handwriting in Children With Developmental Coordination Disorder: A Randomized Control Trial

**DOI:** 10.7759/cureus.78029

**Published:** 2025-01-26

**Authors:** Ruchika J Zade, Moh'd Irshad Qureshi, Raghuveer Raghumahanti, Swadha P Udhoji

**Affiliations:** 1 Neurophysiotherapy, Ravi Nair Physiotherapy College, Datta Meghe Institute of Higher Education and Research (Deemed to be University), Wardha, IND

**Keywords:** developmental coordination disorder, fine motor function, handwriting, leap motion controller, movement assessment battery for children, scale for handwriting evaluation, task-oriented training

## Abstract

Background

The neurodevelopmental disorder, referred to as developmental coordination disorder (DCD), impacts everyday tasks and academic achievement by impairing muscle coordination. DCD children are very often characterized as "clumsy" and "uncoordinated," which can result in easily rectifiable performance difficulties. The aim and objective of the study were to determine the effectiveness of Leap Motion Controller® (LMC®) exercises on fine motor function and handwriting in children with DCD.

Methods

After getting ethical clearance from the university institutional ethics committee (DMIMS(DU)/IEC/2022/897), the Developmental Coordination Disorder Questionnaire (DCD'Q07) questionnaire was given to the parents of the children, and duly filled questionnaires were collected from them after one week. Based on the Diagnostic and Statistical Manual of Mental Disorders (DSM-V) criteria, the children prone to DCD were identified from the questionnaire (n = 30). The participants were subsequently divided into the experimental (n = 15) and control groups (n = 15). Children in the experimental group used the LMC® to perform tasks, while those in the control group completed traditional fine motor exercises for the upper limb. Pre- and post-outcome measures were taken using the scale for handwriting evaluation (SHE) and the movement assessment battery for children (MABC) (manual dexterity) by the assessor who was aware of the outcome measures.

Results

Using software from IBM SPSS Statistics for Windows, Version 27 (Released 2020; IBM Corp., Armonk, New York, United States), descriptive and inferential statistics were performed. The Z-test, Wilcoxon signed-rank test, and Mann-Whitney U test were used for within-group and between-group comparisons. The statistical analysis was conducted with a significance level of p < 0.05. From a statistical and clinical perspective, Group B results were more significant. Improvements in both groups' handwriting and fine motor function were demonstrated by the improvement in the SHE (-0.82, p > 0.412) and MABC (-2.16, p < 0.031) scores.

Conclusion

This study concluded that leap motion technology significantly improved fine motor function and handwriting in children with developmental coordination disorder. Both groups improve children's academic achievement. The study's findings demonstrated the beneficial effects of LMC® on children's handwriting and fine motor skills.

## Introduction

A significant delay in the development of motor coordination, independent of any particular congenital or acquired neurological disease, is what characterizes developmental cerebral palsy. The chronic condition known as developmental coordination deficiency is the primary cause of children's mobility difficulties [1]. These findings emphasize the importance of getting the right help to enhance motor skills and everyday life [2]. The neurodevelopmental disorder, called developmental coordination disorder (DCD), impacts everyday tasks and academic achievement by impairing muscle coordination. DCD children are very often characterized as "clumsy" and "uncoordinated," which can result in easily rectifiable performance difficulties [3]. Orton defined "clumsiness" for the first time in 1937, but the literature did not acknowledge its significance until the early 1960s [4]. Since then, various words have been employed to refer to children whose motor challenges make daily life challenging [5], e.g., clumsy child syndrome [6], perceptual motor dysfunction [7], developmental dyspraxia [8], physical awkwardness [9], and sensory integrative dysfunction [10]. The prevalence rate is estimated to be around 3.8%. The prevalence rate among schoolchildren was 5% in boys and 2.7% in girls [11].

At home, challenges with dressing might appear in tasks requiring fine motor manipulation and organizational abilities, such as managing buttons and zips [12]. Children with DCD struggle in school, mainly when writing, due to a lack of tactile sensation as well as a limited concept of the area; children with DCD can utilize it with skill and speed, and school challenges can be overcome [13]. Hand-eye coordination issues in children with DCD may have an unnoticeable negative impact on their fine motor skills and academic performance. Treatment available to date has focused mainly on gross motor function and coordination. Most of the articles available focus on task-oriented training, motor skills, cognitive orientation to daily occupational performance (CO-OP) [14], virtual reality training, and handwriting training. Evidence has shown that leap motion technology, based on virtual and augmented reality, has profound clinical implications in conditions like this. Leap motion technology has much potential for evaluating and treating hand and finger rehabilitation activities in physical therapy. Hence, this study tries to determine whether the leap motion device enhances fine motor function and handwriting in children with DCD. The aim and objectives of the study were to find out the effectiveness of Leap Motion Controller® (LMC®) exercises on fine motor function and handwriting in children with DCD.

Novel treatment options that could reach people who might not otherwise have access to treatments have been stimulated by recent improvements in the delivery of mental healthcare, which have resulted in the incorporation of numerous technologies [15]. LMC® technology is a small device with infrared sensors and cameras that can only detect a user's hand and finger movements [13]. On the computer screen, the LMC® creates a virtual depiction of the UE and instructs the patient on completing the task [16]. The suggested serious games and the LMC system could be beneficial therapy equipment for enhancing fine motor skills, movement speed, and coordination [17].

Children with DCD often suffer from hand-eye incoordination, which affects their fine motor function. Treatment available to date is mainly focused on gross motor function and coordination. Given the significant prevalence of handwriting problems among children with DCD, strong evidence is required to guide successful assessment and intervention. There is a dearth in the literature which focuses on their fine motor function and handwriting. Thus, there was a strong need to conduct a study which focuses on fine motor function and handwriting in children with DCD.

## Materials and methods

The study recruited children aged seven to 12 who met all four Diagnostic and Statistical Manual of Mental Disorders (DSM-V) criteria and lived in the community. The number of participants was calculated using the Daniel formula [18]. The study included 30 children (15 in Groups A and B). The recruitment process ran from June 2022 to June 2023.

**Figure 1:**
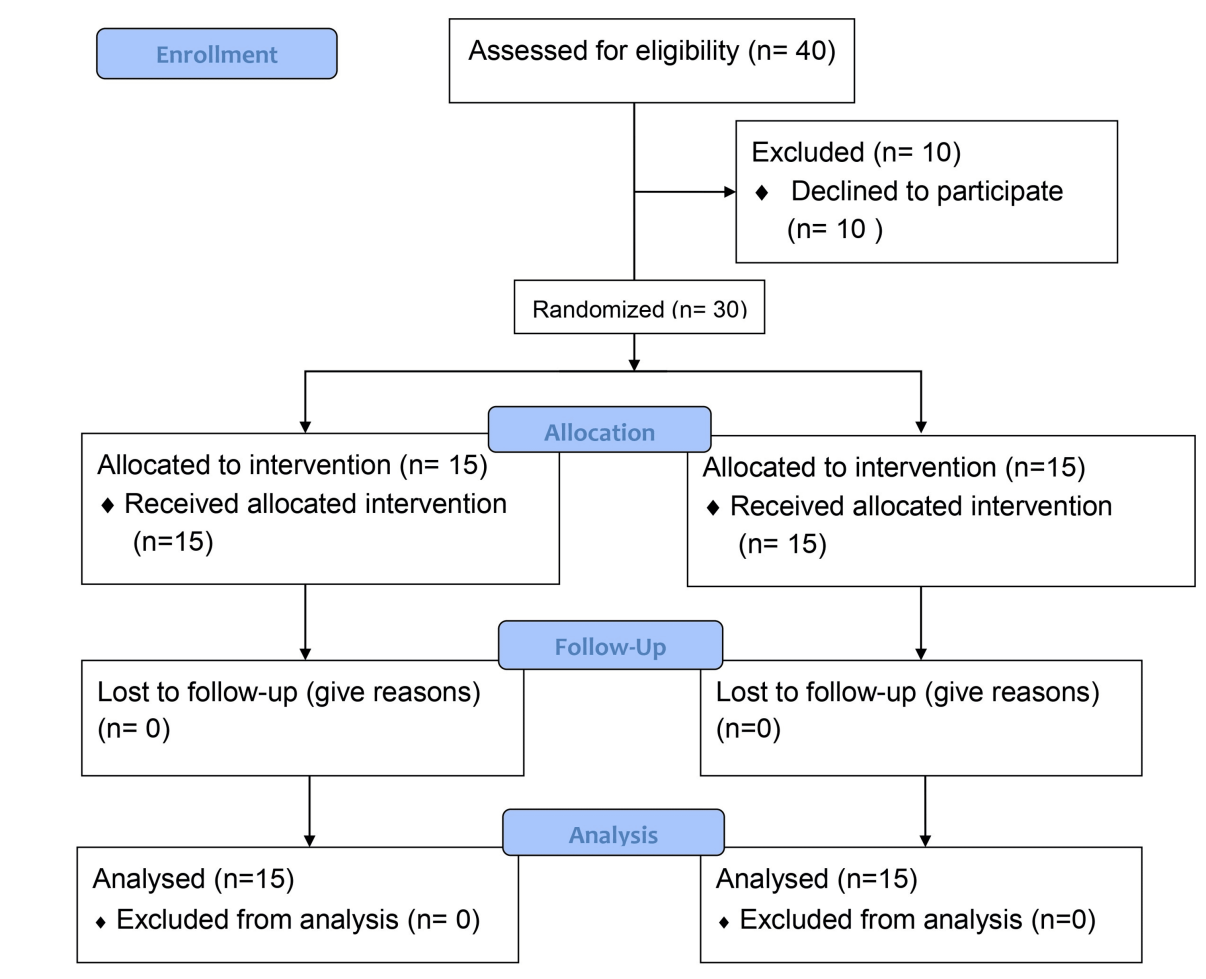
CONSORT flow chart of the study procedure CONSORT: Consolidated Standards for the Reporting of Trials

A total of 40 children were assessed for eligibility. The trial's reports and conduct were standardized through the use of Consolidated Standards for the Reporting of Trials (CONSORT) (Figure 1) [18]. To be taken into consideration, participants have to fulfill the following criteria they have to be between seven and 12 years old and of either gender; have children who are suspected cases of DCD according to the Developmental Coordination Disorder Questionnaire (DCD'Q07); be able to understand and follow instructions; and be willing to participate in the study. Children and parents who did not wish to give consent and children with injuries or fractures of the dominant upper limb were excluded from the study. Every participant gave written informed consent. The number of participants was determined using the Daniel formula [19]. Baseline data is provided, and a total of 30 participants were randomized (15 in each of Groups A and B). The recruitment procedure was carried out from June 2022 to June 2023.

Study design

The institutional ethics committee has approved the study proposal to be carried out at the Ravi Nair Physiotherapy College, Datta Meghe Institute of Higher Education and Research (DMIHER) and Acharya Vinobha Bhave Rural Hospital (AVBRH), Sawangi (Meghe), Wardha. The planned study complied with the ethical standards set by the Central Ethics Committee on Human Research (CECHR), which were decided in the April 2022 meeting. The Institutional Ethics Committee, DMIHER, has discussed and approved the specifics of this planned research study dissertation work for a postgraduate degree course with reference number (DMIMS(DU)/IEC/2022/897). After getting ethical clearance from the university institutional ethics committee, schools in Wardha were identified (a preliminary search was completed, and two schools were positive for research). After obtaining approval from the principals of the respective school, a presentation was given to parents and teachers at AVBRH on the study's relevance and how it might affect the student's academic performance. The DCD'Q07 questionnaire was given to the parents of the children, and duly filled questionnaires were collected from them after one week. Based on DSM-V criteria, the children prone to DCD were identified from the questionnaire (n = 40). Five students who did not meet the inclusion requirements and five who weren't willing to give written consent were excluded. The participants were then divided into the experimental (n = 15) and control groups (n = 15).

Intervention

Children in the experimental group completed activities using the LMC®, while those in the control group completed traditional fine motor exercises for the upper limb. The total study duration was extended to four weeks, where both groups were given therapy for 30 minutes every day, five days a week. Pre- and post-outcome measures were taken using a scale for handwriting evaluation (SHE) and movement assessment battery for children (MABC) (manual dexterity) by the assessor who was aware of the outcome measures and had a similar experience to the physiotherapy resident doing the study. The assessor was blinded for the treatment.

Group A (Control Group)

Grip and release activities were among the tasks which were designed to increase the abilities to flex and stretch the hand as well as the wrist and digit joint range of motion. An increase in several repetitions assisted in progression, initially 10 repetitions; three sets to be performed. The rehabilitation program used various exercise equipment, with varied materials for each sickness group, including skill cubes, Velcro cylinders, therapeutic putties, exercise bands, and objects of various forms, to achieve the goal [20]. Task-oriented training emphasizes the practice of daily tasks as the primary type of intervention (examples include tying shoelaces, zipping, and 15 minutes of handwriting practice) [21].

Group B (Experimental Group)

**Figure 2:**
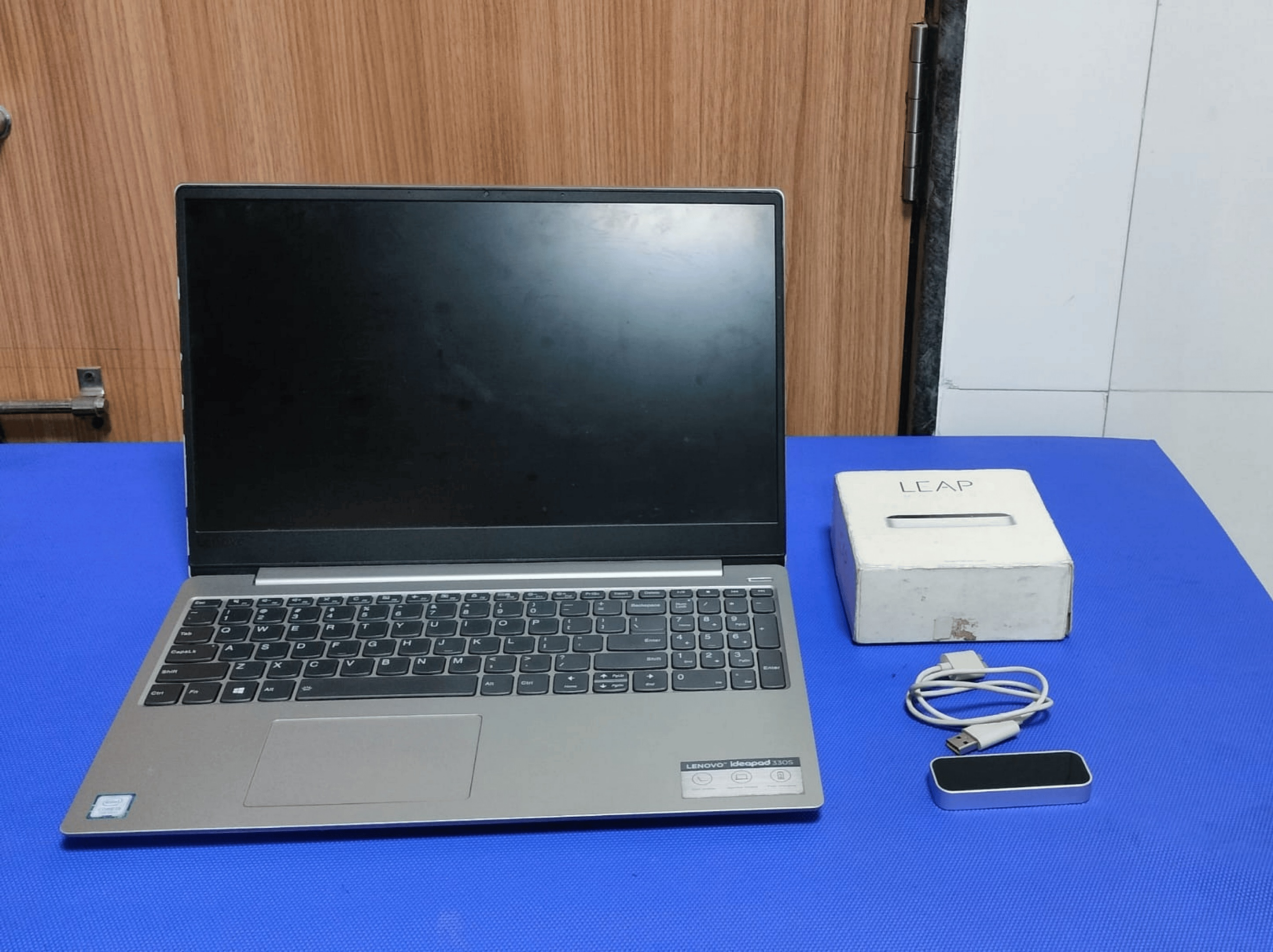
Leap motion device and laptop used to provide virtual reality intervention for experimental group

Both hands were used to play the game, beginning with the dominant hand. Participants should sit in a chair. The LMC® is placed between the body and the LED screen on the table in front of the user. The LMC® used two charged device cameras and three infrared emitters to track any movement of the hands, wrists, and forearms. No marks were necessary because the infrared emitter light reflects off the hands' surface (Figure 2).

Petal-picking game is a game in which the child has to pick up petals. Children should focus on lotus petals in a virtual environment and picking lotus petals in a stimulating environment; a petal-picking game is intended to improve the pinching motor abilities of the fingers. Additionally, the finger's coordination and dexterity were enhanced by this. In the game of "robot-assembling game," the child must pick up blocks. This design was motivated by picking blocks in an engaging setting to improve finger-prehension motor abilities. The primary purposes of the robot-assembly game were to enhance the pronation and supination abilities of the forearm and the pinching motor skills of the fingers [22].

**Figure 3:**
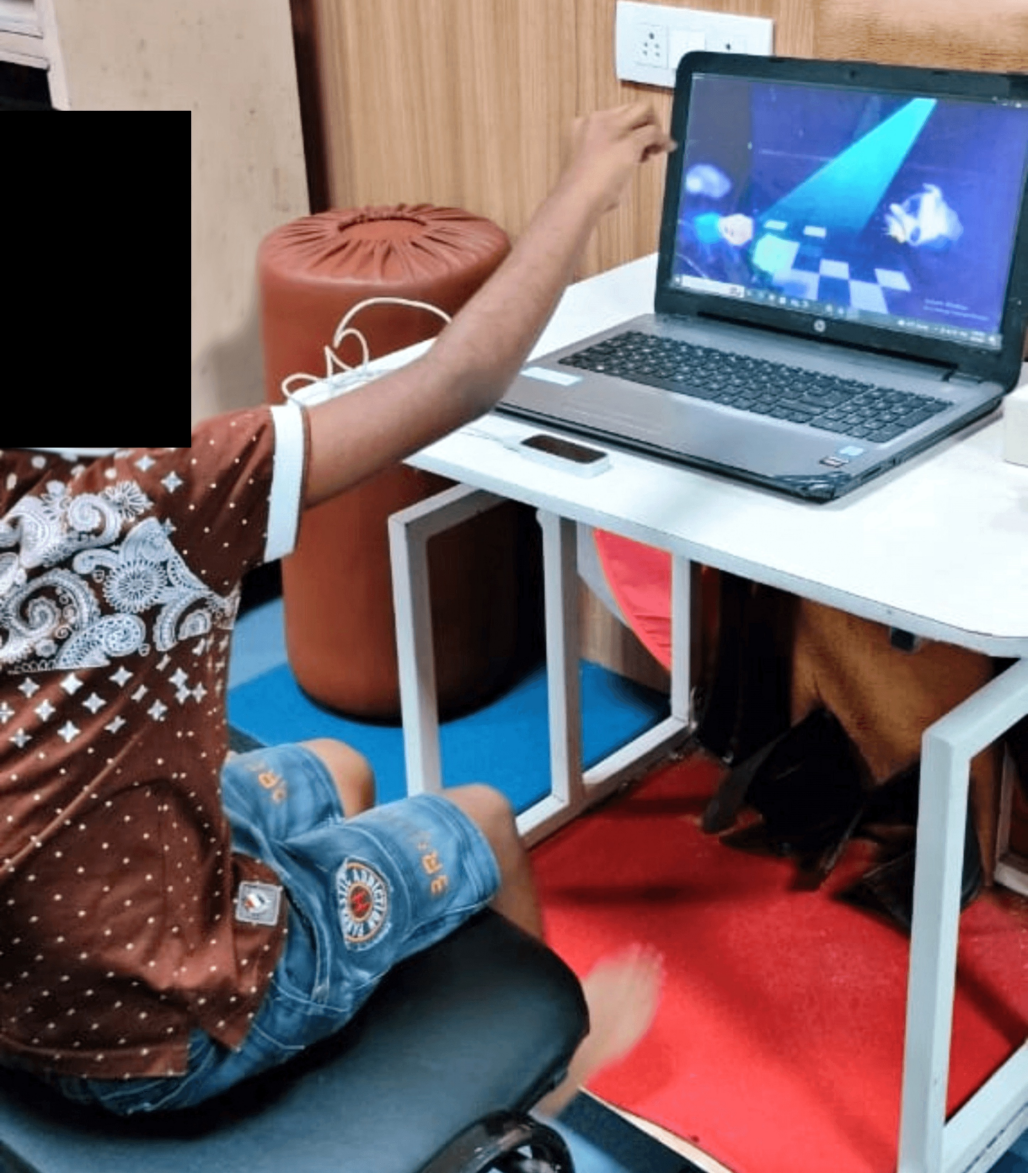
Patient playing with leap motion game (robot-assembling game)

During each session, the primary investigator was sitting beside the participants to provide feedback (if necessary) through verbal, visual, or physical instruction [22,23]. There were three phases to the training protocol. In stage 1, the child received explanations from the physiotherapists about the games, the LMC® sensor, and how to play them; in stage 2, the children were given a chance to test games and the crucial details for proper gameplay (such as the distance between the hand and the sensor) were highlighted; in stage 3, under the direction of physiotherapists, it was made sure that the game has been played appropriately by preventing compensatory motion (Figure 3).

Outcome measure

The DCDQ'07 is a parent-reported questionnaire that assesses and examines children aged five to 15 for developmental coordination deficits [24]. The instrument was developed to assess children's handwriting. This tool can make a substantial difference in the handwriting of typically developing school-age children and those who struggle with motor execution. SHE tool consists of 10 tasks in total. A four-point Likert scale with a minimum of zero and a maximum of three is used to evaluate each task [3]. The most often cited norm-ranked assessment used to identify the existence of DCD in school-aged children is the MABC [14]. The evaluation offers quantitative and qualitative information on how well children performed age-appropriate exercises under manual dexterity, ball skills, and static and dynamic balance [25]. Manual dexterity, a specific MABC component, was included in our study. Three activities were assigned to each participant which included peg placement, lace threading, and trail drawing [26].

Statistical analysis

Forty children were enrolled, and eligibility was determined for each. Ten children were excluded because they failed to fulfill the requirements for inclusion. The trial had 30 patients who complied with the inclusion criteria. Descriptive and inferential statistics were carried out within groups and between groups. The comparison was done using the Z-test, Wilcoxon signed rank test, and Mann-Whitney test with software from IBM SPSS Statistics for Windows, Version 27.0 (Released 2020; IBM Corp., Armonk, New York, United States). For the statistical analysis, the significance level was established at p < 0.05.

## Results

Each of the forty children that were enrolled had their eligibility assessed. Five students who did not meet the inclusion requirements and five who weren't willing to give written consent were excluded. The thirty children in the study who satisfied the eligibility criteria were divided into two groups, A and B. The average age of the patients in Group A was 7-10 years old, while the average age in Group B was 7-9 years old. The gender distribution of the patients was 3:12 for women and 6:9 for men. The chi-square test revealed no statistically significant difference in the children's ages in the two groups (p-value = 0.092). Table 1 provides details about the subjects' baseline characteristics.

**Table 1:** Baseline characteristics NS: not significant

Baseline characteristics	Group A		Group B		p-value
	Mean	SD	Mean	SD	
Age in years	9.13	1.18	8.4	1.18	0.092 (NS)
Gender					
Male	3 (10%)		6 (20%)		0.232 (NS)
Female	12 (40%)		9 (30%)		

The statistical analysis of the outcome measures that were measured, along with the noteworthy variation in values between the groups following rehabilitation, is displayed in Table 2. According to this study, DCD children who used the LMC® device improved their handwriting and fine motor skills. Group B showed a higher improvement in outcome measure scores than Group A.

**Table 2:** Mean MABC and SHE pre- and posttreatment of Groups A and B and intergroup analysis S: significant; NS: nonsignificant; MABC: movement assessment battery for children; SHE: scale for handwriting evaluation

Outcome measure	Group A		p-value	Group B		p-value	Mean difference (X ± SD)		p-value
	Pretreatment	Posttreatment		Pretreatment	Posttreatment		Group A	Group B	
MABC	12.8 ± 1.4	6.46 ± 1.1	0.001 (S)	12.06 ± 1.6	5.46 ± 1.1	0.001 (S)	6.46 ± 1.1	5.46 ± 1.1	0.031 (S)
SHE	14.46 ± 2	22.4 ± 2.3	0.001 (S)	14 ± 2	23.2 ± 2.7	0.001 (S)	22.4 ± 2.3	23.2 ± 2.7	0.412 (NS)

After therapy, both groups showed improvement, but Group B's results were more significant. The Mann-Whitney U test was employed for the intergroup analysis, and the findings for MABC were significant (z-value = -2.161; p-value = 0.031). This suggests that the children's fine motor skills have improved. Children's handwriting improved after treatment, as seen by the comparable outcomes in Groups A and B. Using the Mann-Whitney U-test for intergroup analysis, the findings for SHE were nonsignificant (z-value = -0.82; p-value = 0.412).

## Discussion

This experimental study evaluated the efficacy of leap-motion device training with conventional physiotherapy to enhance handwriting and fine motor function. The MABC and the SHE were used in this study as outcome measures for fine motor skills and handwriting. For the experimental group using the LMC® and the control group using traditional physiotherapy, data were compared within and between groups. The result showed significant differences in all outcomes for within-group comparison and a significant difference in MABC between the groups. Improvements in the conventional group can be attributed to prerequisite skills, without which more complex skills could not emerge, and studies have emphasized the importance of practicing such prerequisites [27]. The SHE and the MABC tools showed substantial differences between the pre- and posttreatment periods in the traditional group. Task-oriented therapies may be helpful for children with DCD to improve mobility test performance [21]. 

Additionally, in the 2018 review study, they aimed to determine the characteristics and effectiveness of motor skill therapy for children with DCD. The authors additionally illustrated how task-oriented approaches, as well as approaches that mix task and process orientations, might be advantageous for kids with DCD [28]. Another study from 2018 found a significant correlation between fine motor abilities and handwriting legibility [29]. The children actively and enthusiastically participated in the intervention, evident through their comments to their parents and active enthusiasm. When operating a virtual environment that requires hand-arm coordination as part of practicing virtual activities. LMC® uses interactive software applications to identify, track, and record finger and hand movements. The LMC® consists of three infrared LEDs, two VGA monochrome cameras, a USB controller, and the LMC® device [13]. On the computer screen, the LMC® provides a virtual representation of the UE and educates the patient on how to complete the task. The LMC® allows users to capture and monitor the precise motions of their hands and fingers [30]. The reason for this was the contralateral sensorimotor cortex's higher activity intensity in the sensorimotor cortex's laterality index [22]. According to a review published in 2022, gesture interaction can be used to improve physical, cognitive, and social skills in controlled virtual environments that mimic real-world situations, which benefits populations with developmental disabilities [31]. These results were in line with those of a 2019 study that found the LMC® can help children who are typically developing, as well as those who do not have DCD or are at risk of developing it. The device can support calligraphy learning or help individuals who are struggling in this school stage, such as adults, children, and older adults who have a writing development delay [17]. These games could serve as rehabilitation tools for Parkinson's patients to improve their coordination, speed of movement, and fine motor dexterity. The serious games for the upper limb (UL) were created by the LMC system to assess the effectiveness of the system and the degree of compliance and satisfaction among patients with mild-to-moderate disease stages [32]. Another 2015 study found that implementing LMC training enhanced the fine motor abilities and cognitive function of children with cognitive and autistic disorders [33]. The statistical analysis for between-group comparisons was done using the Mann-Whitney U test to compare the control and experimental groups. Analysis revealed no difference in the SHE score between groups A and B. Interestingly, the MABC scores between groups A and B showed substantial differences, with the leap motion exercise showing the most improvement compared to the control group. Maximum differences between MABC and SHE were found when comparing MABC and SHE within the experimental group. Thus, compared to the conventional group, the current study's findings and the literature on leap motion exercise show different responses to the variables. LMC® is a practical and efficient haptic virtual reality (VR) technology to enhance certain UE motor functions in patients with neurological conditions. Very low-quality evidence of a significant impact of LMC® compared to conventional exercise on developing fine motor skills in the most affected UE and bilateral central nervous system disorders was demonstrated. In individuals with central nervous system disorders, LMC® had a more significant impact on UE motor function when added to a conventional therapy program, according to the study's findings [32]. Due to impaired internal modeling processes and predictive motor control, children diagnosed with DCD may experience difficulties with motor control. Children with DCD can develop and alter internal movement models, and their predictive modeling has probably improved. Children with developmental disabilities can engage in immersive experiences and learn through virtual reality games [33].

Limitation

Though the study considered the factors that may influence the outcomes, some of those outside the scope of the authors' control might have influenced the results. They include the different etiologies of DCD among children, children having subclinical psychosomatic or learning difficulties coexisting along with DCD, which might have been unnoticed. Further, the study location could only partially control the environmental aspects like ambient noise, light in the surroundings, color of walls, etc. These factors also influence the child's behavior, contributing to the study outcomes. Few children with varying levels of computer knowledge have easy access to technology, which could be used for gaming activities that children find attractive and exciting, influencing their behavior and study outcomes.

Future scope

Future work can involve evaluating additional handwriting recognition software through a digital application employing the LMC® device technology. The ease of use will help to employ this technology in varied settings. Future studies can also examine radiological findings of the brain before and after leap motion technology to understand the neurophysiological implications of this technology in the brain. 

## Conclusions

In this randomized clinical trial, we found that leap motion technology significantly improved fine motor function and handwriting in children with DCD. The SHE intragroup comparison and the MABC inter- and intragroup comparison for fine motor function showed that Group B performed better statistically and clinically significant than Group A. Both groups enhance children's academic achievement. Hence, it can be concluded that LMC® technology can be a practical, interactive approach for rehabilitating fine motor control to enhance handwriting among children with DCD.
